# Publication outcome of abstracts submitted to the American Academy of Ophthalmology meeting*

**DOI:** 10.5195/jmla.2018.314

**Published:** 2018-01-02

**Authors:** Michael Mimouni, Mark Krauthammer, Hamza Abualhasan, Hanan Badarni, Kamal Imtanis, Gilad Allon, Liron Berkovitz, Eytan Z. Blumenthal, Francis B. Mimouni, Gil Amarilyo

## Abstract

**Objective:**

Abstracts submitted to meetings are subject to less rigorous peer review than full-text manuscripts. This study aimed to explore the publication outcome of abstracts presented at the American Academy of Ophthalmology (AAO) annual meeting.

**Methods:**

Abstracts presented at the 2008 AAO meeting were analyzed. Each presented abstract was sought via PubMed to identify if it had been published as a full-text manuscript. The publication outcome, journal impact factor (IF), and time to publication were recorded.

**Results:**

A total of 690 abstracts were reviewed, of which 39.1% were subsequently published. They were published in journals with a median IF of 2.9 (range 0–7.2) and a median publication time of 426 days (range 0–2,133 days). A quarter were published in the journal *Ophthalmology,* with a shorter time to publication (median 282 vs. 534 days, *p*=0.003). Oral presentations were more likely to be published than poster presentations (57.8% vs. 35.9%, *p*<0.001) and in journals with higher IFs (3.2 vs. 2.8, *p*=0.02). Abstracts describing rare diseases had higher publication rates (49.4% vs. 38.0%, *p*=0.04) and were published in higher IF journals (3.7 vs. 2.9, *p*=0.03), within a shorter period of time (358 vs. 428 days, *p*=0.03). In multivariate analysis, affiliation with an institute located in the United States (*p*=0.002), abstracts describing rare diseases (*p*=0.03), and funded studies (*p*=0.03) were associated with publication in higher IF journals.

**Conclusions:**

Almost 40% of abstracts were published. Factors that correlated with publication in journals with higher IF were a focus on rare diseases, affiliation with a US institute, and funding.

## INTRODUCTION

Scientific meetings are a platform for sharing developments in medical research. These meetings allow investigators to present current research data and findings, to exchange ideas, and to initiate future collaboration. Presentation of a study at a scientific conference is a method for rapidly disseminating research information that otherwise would take months or even years until it is made available to the colleagues through peer-reviewed journals [1].

Hundreds, and in some cases thousands, of abstracts are submitted to each scientific meeting. A subset is accepted as an oral presentation, a printed poster, or an electronic poster. The selection process is often not transparent from the author’s perspective. Generally, a program committee reviews the abstracts and decides upon their fates. Often, abstracts that are deemed more original, interesting, and/or of higher scientific value are presented orally, whereas the remaining accepted abstracts are presented as posters.

Not all meeting abstracts eventually get published in a peer-reviewed journal. In fact, previously reported publication rates in other fields of medicine (percentage of abstracts presented in a meeting that were eventually published) range from 20.5% to 68.9% [2, 3]. It is unclear which factors play a role, and to what extent, in the publication outcome of each abstract.

The purpose of this study was to determine the publication rate of abstracts presented at the American Academy of Ophthalmology (AAO) annual meeting and to identify factors associated with (a) higher publication rates, (b) publication in journals with greater impact factors (IFs), and (c) shorter time to publication.

## METHODS

### Abstract data collection

All of the 690 abstracts presented in the 2008 AAO meeting were identified and retrieved from the AAO meeting archive website. The 2008 AAO meeting was chosen to allow a sufficient interval between the time when the abstracts were presented (November 2008) and the time when this study was conducted (December 2014). Some journals consider data greater than 5 years old as obsolete and will not consider papers with old data for review. In addition, according to reports in other fields of medicine, few abstracts are published 5 years beyond their initial presentation [1, 4–6].

Abstracts were categorized by presentation format (poster or oral presentation), ophthalmic subspecialty, research scope (basic science or clinical research), methodology (prospective or retrospective, randomized or non-randomized), prevalence (common or rare disease), and use of a new technique. A rare disease was defined using the definitions specified in the Rare Disease Act of 2002.

### Full-text manuscript search

**Figure 1:**
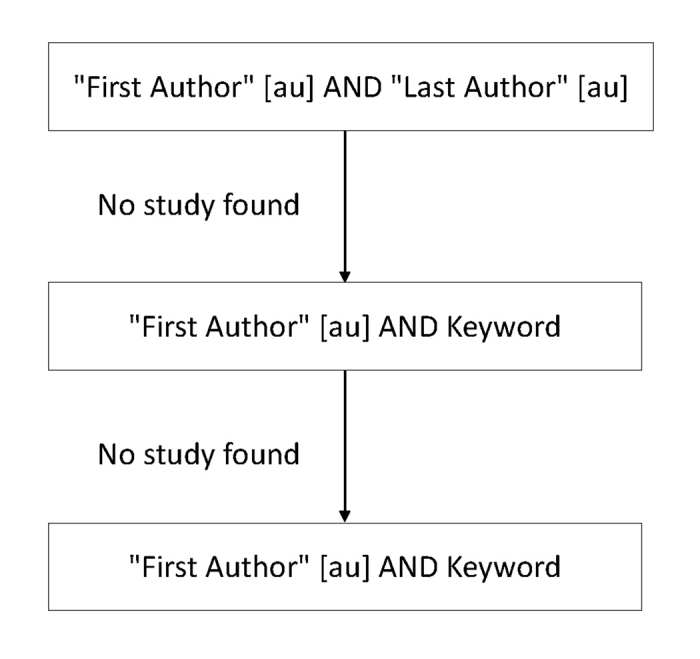
PubMed search algorithm

To identify whether an abstract had been published as a full-text manuscript, a PubMed search was initially conducted. Only AAO abstracts matching full-text manuscripts or case reports appearing in PubMed were categorized as published. AAO abstracts appearing as letters to the editor were not categorized as published. To maximize accuracy, the search protocol included several phases. In the first phase, two independent investigators manually searched the PubMed database for each AAO abstract using a predefined search algorithm composed of a series of queries (Figure 1). The result of each query was manually reviewed for a matching full-text manuscript. A third reviewer adjudicated if disagreement arose.

In the second phase, AAO abstracts that were not found during the first phase were addressed by two coauthors who repeated the search algorithm to verify correct categorization. In the third and final phase, a random sample of AAO abstracts (20%) that had been identified as not published was selected, and the authors were contacted via email (with a second email sent 10–14 days following the first email if it was not answered). The authors were asked to provide information regarding the publication status of their AAO abstracts and any reasons why, in their opinion, it had not yet been published (supplemental appendix).

### Full-text manuscript data collection

For each AAO abstract categorized as published, the following information was collected from the matching full-text manuscript: number of subjects, journal title, journal IF, author’s affiliation, number of authors, funding, and publication date.

### Statistical analysis

Data were analyzed using Minitab software (version 16, Minitab, State College, Pennsylvania). A chi-square test was used for categorical variables. Normality of continuous data was assessed by the Kolmogorov-Smirnov test. The Student *t*-test or one-way analysis of variance (ANOVA) was used for normally distributed data, and the Kruskal-Wallis test for non-normally distributed data. Stepwise regression analysis included independent variables that reached a significance level of <0.05 in univariate analysis. A *p*-value <0.05 was considered statistically significant.

## RESULTS

A total of 690 AAO abstracts were reviewed and analyzed, of which 14.8% (n=102) were oral presentations and 85.2% (n=588) were posters. There was 99.0% agreement (n=683) between the 2 independent investigators regarding the publication status of each abstract. The overall publication rate was 39.1% (n=270).

An email was sent to the authors of 20% (randomly sampled) of the AAO abstracts identified as not having been published (n=84), and a response was received regarding 26 AAO abstracts (31% response rate). Twelve of the authors replied that the full-text manuscript was rejected and resubmission abandoned due to low likelihood of acceptance (n=6), they had no time to resubmit (n=2), or other reasons (n=4). Seven authors replied that a different version of the data was published following rejection of the original study data. Four authors replied that the full-text manuscript was never submitted because they did not have time (n=2) or because the study was ongoing (n=2). Two authors replied that they had insufficient recollection of the abstract and its publishing outcome. One author replied that the full-text manuscript was published based on data presented in the meeting. Therefore, 1 out of 26 abstracts was found to be incorrectly categorized as not being published (3.8%), following the authors’ responses.

The number of AAO abstracts presented in each subspecialty and the corresponding publication rate are presented in Table 1. Briefly, there was a significant difference in publication rate between subspecialties (χ^2^=23.85, *df*=10, *p*=0.008), with intraocular inflammation and uveitis demonstrating the highest publication rate and refractive surgery the lowest publication rate.

**Table 1:** Distribution of abstracts presented and publication rate depending on subspecialty

Subspecialty	Number	Percent	Publication rate
Intraocular inflammation and uveitis	23	(3.3%)	52.2%
Cornea, external disease	107	(15.5%)	50.5%
Ocular tumors and pathology	20	(2.9%)	50.0%
Pediatric ophthalmology, strabismus	31	(4.5%)	48.4%
Neuro-ophthalmology	22	(3.2%)	45.5%
Orbit, lacrimal, plastic surgery	34	(4.9%)	44.1%
Glaucoma	107	(15.5%)	42.1%
Retina, vitreous	163	(23.6%)	36.8%
Others	21	(3.0%)	33.3%
Cataract	95	(13.8%)	27.4%
Refractive surgery	67	(9.7%)	23.9%

The univariate analysis of factors associated with higher publication rates of AAO abstracts is depicted in Table 2. Oral presentations had a significantly higher publication rate than poster presentations. Similarly, abstracts describing rare diseases had a significantly higher publication rate than abstracts describing common diseases.

**Table 2:** Differences in publication rates of abstracts based on various factors

Parameter	Publication rate (%)	χ^2^	p*
Oral (n=102) vs. poster (n=588) presentation	57.8% vs. 35.9%	17.16	<0.001
New (n=144) vs. established (n=546) technique	38.9% vs. 39.2%	0.01	0.95
Case (n=23) vs. non-case (n=666) report	52.2% vs. 38.7%	1.68	0.19
Basic science (n=45) vs. clinical research (n=644)	44.4% vs. 38.8%	0.56	0.46
Prospective (n=317) vs. retrospective (n=368)	38.8% vs. 39.4%	0.03	0.87
Randomized (n=170) vs. non-randomized (n=514)	37.1% vs. 39.9%	0.43	0.51
Rare (n=79) vs. common (n=606) disease	49.4% vs. 38.0%	3.82	0.04
Sample size >1,000 (n=41) vs. ≤1,000 (n=607)	41.5% vs. 39.4%	0.07	0.79

* Chi-square.

Table 3 depicts the results of stepwise binary logistic regression. Briefly, AAO abstracts were more likely to be published if they were orally presented, and the subspecialty of the presented abstract remained a significant predictor of whether it would be subsequently published.

**Table 3:** Multivariate binary logistic regression analysis of factors predicting publication

Parameter	R^2^	Odds ratio	95% Confidence interval (CI)	*p*
Oral presentation	2.6	2.9	1.8–4.5	<0.001
Subspecialty	2.2	—	—	0.007
Rare disease	0.3	1.6	0.9–2.6	0.10

The median IF of journals in which abstracts were published was 2.9 (range 0–7.2). Of those published, 25.2% were published in the journal *Ophthalmology* (n=68), which is the official journal of the AAO. Table 4 shows the median IFs of the journals in which AAO abstracts were published, based on the factors examined. Briefly, oral presentations, abstracts describing rare diseases, studies with more than 1,000 subjects, funded studies, and studies in which the first author was affiliated with an institution in the United States were published in journals with higher IFs than those of their counterparts.

**Table 4:** Univariate analysis of factors and the impact factor (IF) of the journal in which they were published

Parameter	Median IF (range)	H-value	p*
Oral (n=59) vs. poster (n=211) presentation	3.2 (0–6.7) vs. 2.8 (0–7.2)	5.84	0.02
New (n=56) vs. established (n=214) technique	2.5 (0–6.2) vs. 2.9 (0–7.2)	0.66	0.36
Case (n=12) vs. non-case (n=258) report	2.0 (0–5.5) vs. 2.9 (0–7.2)	1.00	0.30
Basic science (n=20) vs. clinical research (n=250)	3.6 (0–6.2) vs. 2.9 (0–7.2)	3.93	0.06
Prospective (n=123) vs. retrospective (n=145)	2.8 (0–7.2) vs. 2.9 (0–6.2)	0.22	0.58
Randomized (n=63) vs. non-randomized (n=205)	3.0 (0–6.7) vs. 2.9 (0–7.2)	0.25	0.69
Rare (n=39) vs. common (n=230) disease	3.7 (0–6.2) vs. 2.9 (0–7.2)	4.50	0.03
Sample size >1,000 (n=17) vs. ≤1,000 (n=239)	4.3 (1.37–7.2) vs. 2.9 (0–6.7)	6.32	0.01
Funded (n=75) vs. non-funded (n=157)	3.2 (1.37–7.2) vs. 2.8 (0–6.2)	6.08	0.02
US (n=120) vs. non-US (n=149) affiliation	3.2 (0–6.2) vs. 2.5 (0–7.2)	5.56	0.01
† Subspecialty	—	31.51	0.003

* Kruskal-Wallis.

† Pediatric and glaucoma AAO abstracts were published in journals with the highest (5.27) and lowest (1.98) median IF, respectively.

Ranked stepwise multiple regression analysis showed that abstracts with a first author affiliated with an institution in the United States (R^2^=4.2, *p*=0.002), those that described rare diseases (R^2^=2.3, *p*=0.03), and funded studies (R^2^=2.1, *p*=0.03) were associated with publication in journals with higher IFs.

The median publication time was 426 days (range 0–2,133 days). Meeting abstracts that were published in *Ophthalmology* were published nearly twice as fast as those that were published elsewhere (median 282 vs. 534 days, *p*=0.003). Table 5 depicts the time to publication depending on different factors. Briefly, studies describing rare diseases were published faster than those describing common diseases, and studies in which the number of subjects in the full-text manuscript was larger than that in the abstract (“subjects increased”) had longer times to publication. Both factors remained significant in ranked stepwise multiple regression analysis (subjects increased, R^2^=2.0, *p*=0.02; rare disease, R^2^=1.8, *p*=0.04).

**Table 5:** Univariate analysis of factors and their publication times

Parameter	Median publication time in days (range)	H-value	p^*^
Oral (n=59) vs. poster (n=211) presentation	388 (0–1,819) vs. 450 (0–2,133)	0.21	0.54
New (n=56) vs. established (n=214) technique	478 (0–1,887) vs. 421 (0–2,133)	1.73	0.23
Case (n=12) vs. non-case (n=258) report	450 (0–1,546) vs. 426 (0–2,133)	0.23	0.61
Basic science (n=20) vs. clinical research (n=250)	326 (0–1,518) vs. 450 (0–2,133)	2.18	0.24
Prospective (n=123) vs. retrospective (n=145)	438 (0–2,133) vs. 426 (0–2,061)	0.33	0.41
Randomized (n=63) vs. non-randomized (n=205)	478 (0–2,133) vs. 425 (0–2,031)	0.58	0.35
Rare (n=39) vs. common (n=230) disease	358 (0–1,758) vs. 428 (0–2,133)	4.60	0.03
Sample size >1,000 (n=17) vs. ≤1,000 (n=239)	419 (23–2,133) vs. 464 (0–2,031)	0.00	0.98
Funded (n=75) vs. non-funded (n=157)	387 (0–2,133) vs. 426 (0–2,031)	0.11	0.66
US (n=120) vs. non-US (n=149) affiliation	427 (0–2,133) vs. 425 (0–2,031)	0.13	0.73
Subjects increased (n=86) vs. no increase (n=170)	570 (0–2,031) vs. 388 (0–2,133)	6.44	0.02
Subspecialty	—	8.25	0.56

## DISCUSSION

This study analyzed the publication outcomes of 690 abstracts presented at the AAO 2008 annual meeting. The overall publication rate of 39.1% was comparable to the reported publication rates of scientific meetings in other fields of medicine [2, 3, 7, 8] and the Canadian Ophthalmological Society annual meeting (45.7%) [9], while higher than that of the Royal College of Ophthalmologist annual congress (26.6%) [10]. However, it is strikingly lower than a study published more than 2 decades ago reporting that 57% of the AAO meeting abstracts reached full-text manuscript publication [6].

In the current study, most abstracts presented in the AAO meeting were not subsequently published in a peer-reviewed journal, either because they were never submitted to a journal or did not withstand the peer-review process. Interestingly, Saldanha et al. analyzed randomized controlled trials that had been presented at the 2001–2004 Association for Research in Vision and Ophthalmology conferences and reported that more than half the examined publications exhibited some amount of discordance in the main outcome results when compared with presented abstracts, calling into question the dependability of conference abstracts [11]. Together, these findings suggest that researchers should interpret preliminary data presented at scientific meetings with caution.

In this study, oral presentations were more likely to be published than poster presentations, a finding that was supported by a previous study in the fields of rheumatology [7] and veterinary ophthalmology [12]. Only a subset of AAO abstracts (14.8% in 2008) were selected to be presented in oral format. According to the AAO website, the Annual Meeting Program Committee chooses submissions to be presented as papers (i.e., oral presentations) based on originality, clinical relevance, comprehensiveness, and potential as a stimulus for prepared discussion, giving greater priority to novel work [13]. As such, it was not surprising that oral presentations go on to be published more often than poster presentations.

The median IF of journals in which AAO abstracts were published was 2.9, well above that of the 1.7 median IF of ophthalmology journals at the time this study was conducted (Journal Citation Reports 2013). Although IF has limitations, it is still considered a legitimate indicator of journal quality [14, 15]. The relatively high IF of the published AAO abstracts might be, as previously mentioned, the result of a filtering process of the AAO program committees in which the finest abstracts were chosen.

Interestingly, AAO abstracts in which the first author was affiliated with an institution in the United States were published in journals with higher IFs. This was supported by a previous study analyzing abstracts from the European College of Veterinary Ophthalmologists meeting, where author nationality and academic association were found to be significant factors of publication [12]. This might be the result of US study groups being based in tertiary centers with higher funding and more abundant resources available for conducting high-quality research. In addition, the English language serves as the main language for a majority of the scientific journals in general and ophthalmology journals specifically. Therefore, native English speakers may have an advantage when it comes to scientific writing and publishing. Indeed, non-native-English-speaking academicians have been reported to be less satisfied with the peer-review process [16] and may benefit from scientific writing training programs [17].

Funded studies were published in journals with a higher IF than non-funded studies. This may be the result of a filtering process performed by the parties providing the funding, in which only the studies deemed worth investigating are granted financial support. In addition, the funding of a study provides the researcher with ampler resources than those that do not receive funding. Finally, a study that has received funding is generally supervised by the funding party, who would have a vested interest in verifying that the study and its findings are indeed published. The financial relationship between pharmaceutical companies and researchers has increased dramatically and has been the subject of much debate [18, 19]. It has been shown that regardless of the main outcome results, conflicts of interests of first authors of abstracts are associated with whether they go on to be published [11].

Studies of rare diseases were published in journals with higher IFs and with shorter times to publication. This finding is encouraging considering that for many rare diseases, there are no effective treatments [20]. We must note that nearly one-third of “transformative drugs,” defined as pharmaceuticals that are innovative and have groundbreaking effects on patient care, that were approved between 1984 and 2009 were originally developed for rare diseases before broader applicability was found [21]. Therefore, rare diseases are not only more interesting for journal editors, reviewers, and readers, they at times constitute a bridge for scientific breakthroughs that later apply to more common diseases.

Median publication time was 426 days, similar to that of abstracts presented in a previous study in the field of rheumatology [7]. Those published in *Ophthalmology* were published in half of the time of those published elsewhere. This may be a byproduct of the AAO meeting and *Ophthalmology* journal guidelines stating that *Ophthalmology* has right of first refusal on these manuscripts. This may have also been the result of the relatively short time to publication of *Ophthalmology*, which was recently reported to be 297.5 days (range 266.3–353.0 days) [22], similar to the median of 282.0 days in this study. Also, studies in which the number of participants increased between the time of the AAO abstract and the full-text manuscript had a longer time to publication. This can be explained by the additional amount of time and resources that would be required to include more subjects in a study.

One limitation of this study is that meeting abstract data and findings may be partial or preliminary and the results may vary greatly before being submitted for publication. For instance, this study has shown that the number of study participants might increase. As such, many additional factors could change that we may have overlooked, such as diagnostic accuracy [23] and author conflict of interest [11]. In addition, the multiple tests performed in this study could potentially have led to capitalizing on chance (i.e., type I error).

Another limitation of this study relates to the fact that the data from a single meeting were analyzed and, therefore, might not apply to AAO meetings from other years or to other ophthalmic scientific meetings. Further studies could be performed to compare the publication rates of AAO meetings with those of other international ophthalmology meetings. An additional limitation is that we were unable to follow-up with abstract proposals that were not accepted for the conference, as it was possible that some of them were ultimately published as well. Lastly, despite the rigorous attempt to correctly identify the publication outcome of each abstract, the questionnaire sent to the authors showed that 4% were falsely identified as not published.

This study shows that less than half of the abstracts accepted for presentation at an AAO annual meeting were eventually published. Those that were published appeared in peer-reviewed journals with relatively high IFs. This may reflect that abstracts accepted for presentation by the AAO program committees, particularly as oral presentations, are a well-selected group of research studies.

## Supplemental File

Appendix Abstract author contact email
