# Supplementary material for: Publication outcome of abstracts submitted to the American Academy of Ophthalmology meeting
Source: J Med Libr Assoc. 2018 Jan 2;106(1):57–64. doi: 10.5195/jmla.2018.314 (PMC5764594; doi:10.5195/jmla.2018.314)
Supplement: Appendix [file jmla-106-57-s001.pdf]

Journal of the Medical Library Association

Supplemental content to
J Med Libr Assoc. Jan;106(1):dx.doi.org/10.5195/jmla.2018.314
www.jmla.mlanet.org
© Mimouni, Krauthammer, Abualhasan, Badarni, Imtanis, Allon, Berkovitz, Blumenthal, Mimouni, Amarilyo 2018

Publication outcome of abstracts submitted to the American Academy of Ophthalmology meeting

Michael Mimouni, MD; Mark Krauthammer, MD; Hamza Abualhasan, MD; Hanan Badarni, MD; Kamal Imtanis, MD; Gilad Allon, MD; Liron Berkovitz MD; Eytan Z. Blumenthal, MD; Francis B. Mimouni, MD; Gil Amarilyo, MD

APPENDIX

Abstract author contact email

Dear Dr. _______,
Following an analysis of abstracts presented at the 2008 American Academy of Ophthalmology (AAO) annual scientific meeting, we, an independent study group, believe that the data that your group presented did not result in a published full-length manuscript.

The title of the abstract your group submitted was:

As part of a study that we are conducting we would like to assess the factors that may have interfered with the publication of the data that you presented at the meeting.

Please note that this two-question length questionnaire is completely voluntary and strictly confidential.

1. What is the status of the abstract data that you presented at the 2008 AAO annual scientific meeting?
   a. Published as full-length manuscript
   b. In press
   c. Under consideration
   d. Submitted/Rejected: If so, are you planning on resubmitting to a different journal?
   e. Not submitted
   f. Cannot recall presented abstract
   g. Other

2. If the abstract data have not been published or submitted/resubmitted for publication as a full-length manuscript, what is the main reason for this decision?
   a. Did not have sufficient time to prepare manuscript
   b. Study is still ongoing
   c. A coauthor is responsible for writing and submitting the manuscript
   d. Low likelihood of acceptance
   e. Different version of data published
   f. Other

Thank you for participating in our questionnaire investigating "Publication Outcomes of 2008 AAO Annual Meeting Abstracts."

JMLA Journal of the Medical Library Association

SC

Supplemental content to
J Med Libr Assoc. Jan;106(1):dx.doi.org/10.5195/jmla.2018.314
www.jmla.mlanet.org
© Mimouni, Krauthammer, Abualhasan, Badarni, Imtanis, Allon, Berkovitz, Blumenthal, Mimouni, Amarilyo 2018

If you have any questions or concerns regarding this study, please feel free to contact me personally.

Sincerely,
Michael Mimouni, MD
Ophthalmology Resident
Rambam Health Care Campus
Haifa, Israel
m_mimouni@r

P.S.: You may opt not to participate and in the case you do choose to participate, your confidentiality: your name will not appear on these surveys and all potential personal identifiers—including address, affiliation, and IP address—will not be collected. There are no known risks nor any direct benefits associated with your participation. You will not be compensated for your participation. Interview/survey information will be locked in a cabinet or password protected in a secure location. By participating in the survey, you are giving permission for the investigators to use collected information for research purposes. This survey is not an official survey of the AAO but instead is conducted by an independent study group.
